# Direct Parent Engagement to Improve Fundamental Movement Skills in Children: A Systematic Review

**DOI:** 10.3390/children10071247

**Published:** 2023-07-19

**Authors:** Robert J. Flynn, Andy Pringle, Clare M. P. Roscoe

**Affiliations:** Department of Sport and Exercise Science, Clinical Exercise and Rehabilitation Research Centre, University of Derby, Kedleston Road, Derby DE22 1GB, UK; rob_fly10@hotmail.co.uk (R.J.F.); c.roscoe@derby.ac.uk (C.M.P.R.)

**Keywords:** fundamental movement skills, physical activity, parent engagement, children, interventions, smartphone apps

## Abstract

Fundamental movement skills (FMS) are basic movements in children that represent the building blocks for more complex motor skill development and act as a prerequisite for enduring sport and physical activity (PA) engagement and positive health-related behaviours. The FMS proficiency is currently inadequate worldwide, and consequently there are alarming levels of inactivity and childhood obesity. However, parents are role models to their children and possess the power to influence their PA behaviour. This review investigated if parent-focused interventions could improve FMS in 2–7-year-old children and evaluated which setting and method of parent engagement was most impactful. Keyword searches were conducted via Scopus, Web of Science, SPORTDiscus, PubMed, Science Direct, and Google Scholar. Only nine articles met the inclusion criteria. No research originated from the United Kingdom, highlighting the urgent need for further FMS interventions involving parents. The FMS improved in all nine studies, with significant changes in seven of the articles (*p* < 0.05). Parent–child co-activity, the education and empowerment of parents, and the provision of clear FMS guidance, messaging, and structure can positively influence children’s FMS. Recently, smartphone apps have increased the feasibility and accessibility of FMS practice at home and may be integral to future interventions. Further research with direct parental involvement is clearly warranted.

## 1. Introduction

Fundamental movement skills (FMS) are the basic abilities of a child to execute simple movements and proficiencies that provide the building blocks for the normal development and maturation of more complex motor skills [1,2]. The FMS can be categorised into locomotor skills that consist of running, jumping, hopping, and galloping; object control skills such as throwing, rolling, catching, and kicking; and postural control movements involving bending, twisting, and body rolling [3]. Children obtain and cultivate gross and fine motor skills at a prolific rate early in their lives, rendering early childhood (2–5 years) a critical period for the acquisition of FMS and the progression of overall physical literacy [4,5]. The FMS also act as a prerequisite for daily functioning, sports, and physical activity (PA) engagement in later life [2].

A conceptual model of engagement has proposed that a reciprocal relationship exists between FMS, PA, and health-related fitness [6]. The model explores how FMS prove vital in the initiation, maintenance, or deterioration of PA; an assertion that has been vigorously investigated and acknowledged by many subsequent studies [7]. Indeed, FMS proficiency appears to provide the motivation and confidence to maintain individually appropriate PA levels, which in turn creates a positive synergistic trajectory that enhances physical, psychological, and cognitive wellbeing and establishes positive health behaviours that endure throughout life [8,9,10]. Conversely, children with inadequate FMS ability enter a negative spiral of disengagement that yields less PA and poor health-related fitness [11,12].

Children should be capable of mastering FMS by the age of 7 years as they begin to engage in more specialised skills associated with sports and exercise [13]. However, the motor proficiency in children around the world is rated as below average, while studies have suggested that the FMS of children in the United Kingdom (UK) are inadequate [4,5,13]. In similar research, less than 20% of 492 children aged 6–9 years from within key stages 1 and 2 of the English school system were fully competent in four key FMS identified by the physical education (PE) curriculum [14]. Comparatively, an Irish study that comprised 242 adolescent children aged 12–13 years discovered that a mere 11% were proficient or nearly proficient in the nine FMS that were measured [15]. The evidence presented here is alarming and further investigation of FMS in children is urgently required.

The current UK guidelines recommend that preschool children up to the age of 5 years should aim to achieve a minimum of 180 min of PA per day, including 60 min of moderate to vigorous PA (MVPA) [16,17]. Comparatively, the World Health Organization (WHO) advises children aged 5 years and over should complete at least 60 min of MVPA daily [18]. However, the domestic and global PA participation rates are unacceptably low [19,20]. Only 10% of UK preschool children and 24% of American children aged 6–17 years exercise enough daily, while 80% of adolescents worldwide are classed as inactive [19,21,22,23]. Approximately 88% of children with unsatisfactory motor competence failed to achieve their recommended daily activity [24]. This is a concern to preschool, primary, and secondary aged children, and suggests that FMS and PA interventions are required.

Notably, poor FMS and low PA participation in children and adolescents are inversely associated with weight status [9]. Children who display poor movement competency tend to participate in less PA and are more likely to gain weight, creating a vicious cycle where the weight gain itself restricts future PA participation and FMS practice [25,26]. Physical inactivity is considered to be one of the major contributors towards an ever-growing childhood obesity epidemic and its associated health consequences, such as type II diabetes mellitus and cardiovascular diseases [27]. It has been estimated that 40 million preschool children are currently overweight or obese worldwide [28]. Disturbingly, the children of this generation are expected to die younger than their parents for the first time in modern history [29], and this highlights the critical need to develop a greater understanding of FMS and how to better engage children in positive PA behaviours.

An influential factor on FMS ability and PA engagement appears to be gender [30]. Girls generally outperform boys in terms of fine motor ability, balance, and locomotor skills, whereas boys are commonly superior in their gross motor ability and object control [5,30]. However, these patterns are not consistently observed, possibly because the brain structure and development differ between the sexes in the early years; therefore, the FMS development is not uniform amongst preschoolers when analysing them by age [31]. With that said, girls in particular can be negatively influenced by socialisation and environmental factors that reduce PA and FMS practice [32,33,34]. Young girls tend to interact in a caring and shared manner, which contrasts with the competitive and egocentric traits displayed by boys [35]. These traits may in turn influence game choices and play interactions, with girls favouring dance and role play and boys showing preference for ball games [36].

Studies have highlighted that children of low socioeconomic status are less likely to be proficient in FMS, less active, and to have insufficient cardiorespiratory fitness when compared to their counterparts of higher status [37]. The reasons for this are multifactual and interrelated and include poor knowledge of FMS and PA; a lack of facilities, opportunities, and safety in the neighbourhood; language barriers; and high volumes of screen-time [38,39,40]. Furthermore, certain cultures may obstruct female PA participation as they view sport and exercise as being primarily of the masculine domain [41]. Interventions should, therefore, show specificity and consider gender, age, culture, and status factors to successfully develop FMS and PA programmes [4,5,31,38].

Schools in the UK offer the means to support FMS and PA in primary-aged children via the national curriculum and through the provision of equipment, facilities, and personnel [9]. FMS development is reliant on access to coaching, feedback, and practice [8,15]. It has been postulated that children whose errors are corrected during FMS training show greater improvement than children who are left uncorrected [42]. However, a lack of school funding over a 10-year period has reduced the quality of physical education (PE) lessons, and as few as 15% of educators currently possess sufficient knowledge and the capability to deliver effective FMS guidance and assessments [43]. This is compounded by the absence of a curriculum for children under the age of 5 years in the UK, leaving teachers who lack the confidence to teach FMS without the relevant guidance to deliver adequate FMS practice during the most critical stage of children’s development [44].

Despite the recent issues related to the school-based delivery of FMS, a compelling body of evidence has presented promising outcomes in children’s motor competency and PA behaviour through school-based interventions [33,45,46]. A recent randomised controlled trial highlighted the importance of fun during PE lessons to improve FMS competency more effectively in primary school children [45]. Equally, studies have encouraged and listened to the student voice and incorporated game-based approaches, helping to make PA more stimulating, age-appropriate, and purposeful within an educational context [47,48,49]. Improvements in FMS through interventions in the school setting have also been shown to have a mediating effect on MVPA engagement [50]. However, a lack of follow-up studies, the risk of assessment bias, the challenge of monitoring PA in young children, and a reliance on parental reporting have raised questions against the validity and sustainability of such interventions [16,33].

Another key research area has been within childcare [2]. Preschools and childcare centres provide quality provisions for engaging activity via access to outdoor spaces and equipment [51]. However, preschoolers’ PA opportunities are often restricted by the rigidity of playground regulations due to the perception of risk and the lack of early childhood teacher programmes allocated to PA support [8,52]. Nevertheless, studies have reported improvements in FMS and PA engagement through interventions that have enhanced the knowledge and self-efficacy of childcare providers and via the implementation of mandatory government policies and capacity-building initiatives targeting PA in childcare settings [53,54,55]. Therefore, it seems apparent that more promotion of FMS interventions will not only improve FMS competency but also the PA levels and health-related outcomes in preschool children.

The school and childcare domains have thus far been the primary focus of research and interventions, whereas community and home programmes have received less attention despite children spending approximately half of their days throughout the year at home [56,57,58]. Sedentary behaviour is considered more likely to occur at home, with a recent UK study of preschool children reporting significantly greater volumes of sedentary time on weekends (96.9%) compared to weekdays (91.9%) (*p* < 0.05) and less time in MVPA at weekends (2.0%) than during the week (6.3%) [59]. For this reason, out of school periods should be considered vital windows and targeted by PA interventions [9], with it being critical to actively involve parents in FMS programmes to provide them with the appropriate skills and strategies that can be implemented at home [60]. Parents have the power and influence to provide their children with a supportive environment, equipment, and the freedom to move, essentially serving as “gate keepers”, as they are the main influence on their child’s behaviour and PA opportunities [61,62]. This is demonstrated by the positive association between parents who place a high value on sport and exercise and the subsequent active lifestyle of their children [63], whereas solicitous and overprotective parents inadvertently restrict outdoor activity that yields weaker FMS development in their children [64]. FMS practice is further inhibited by parents who frequently overestimate their children’s PA levels, inaccurately perceive their FMS ability, or are simply unaware of FMS and PA guidelines [38,65,66]. Consequently, the children from these families are often not active enough and the parents fail to recognise the need to encourage more active behaviour [67,68]. Therefore, raising the parental awareness of PA guidelines, policies, and FMS may be an important first step in family interventions [65,69]. Equally important may be the education of parents in FMS performance and skill perception to empower them to become role models, which could enhance the FMS proficiency and PA levels of children at home [66,67,70]. One method suggested to achieve this is through specialist-led interventions taught in conjunction with parent participation [71]. Community interventions can increase parent confidence, motivation, and knowledge of physically active behaviours and physical literacy, which in turn encourage self-efficacy to apply learnings at home with their children [72]. The co-activity of parents and children has been postulated to be the most advantageous method of improving FMS in children [73], with an intervention involving fathers’ co-activity with their children successfully increasing the PA volume of both parent and child and FMS competence in the children [28,74]. Comparatively, an innovative and novel method of delivering FMS education and guidance to the parent in the home environment without the requirement of specialist support is via digital and mobile applications [75]. These platforms are reported by parents to be user friendly and have created easy-to-deliver, parent-led curricula, which have produced substantial improvements in the FMS proficiency of preschool children over a short period of time [76,77].

Based on the literature, the primary aim of this study is to investigate if PA interventions that directly involve the parent or guardian can elicit improvements in FMS in 2–7-year-old children. The further aims are to explore interventional settings and methods to establish a recommendation as to which form of parental engagement is most effective for motor competence in children.

## 2. Materials and Methods

### 2.1. Protocol and Registration

The details of this systematic review were registered with PROSPERO in November 2022. The review protocol is available on the PROSPERO website by searching the registration number CRD42022370921 or via the following address: https://www.crd.york.ac.uk/prospero/display_record.php?RecordID=370921 (accessed on (accessed 13 June 2023)).

### 2.2. Study Selection Criteria

An exhaustive systematic literature review was conducted in accordance with the Preferred Reporting Items for Systematic Reviews and Meta-Analyses (PRISMA) framework to assemble all English language (or translated), peer-reviewed articles published worldwide between January 2012 and November 2022. All studies that examined the influence of PA interventions with direct and explicit parental participation on the FMS proficiency of their early years children, either within the home environment, the community, or the childcare and educational settings, were considered. The determination of what qualified as “direct and explicit” parental participation was subjective. Studies that made the parents the main focus of the intervention, involved joint parent–child participation, or comprised parent education in conjunction with the provision of training programmes or manuals for parents to deliver to their children were reasoned to be explicit involvement. Indirect parent involvement such as handouts, newsletters, storybooks, and music CDs were insufficient for the purposes of this review. These determinations were based on the literature, which recommended that parent involvement must extend beyond educational handouts to provide parents with adequate capacity to support their children with motor competency [60,78,79].

Any study designs were deemed suitable providing they clearly reported quantitative pre- and post-data for at least one component of FMS proficiency (locomotor, object control, or balance). Ideally, both intervention and control groups should be present within the research to act as a point of comparison and to add validity to the findings. However, due to a lack of research in this area, studies were included even if a control group was absent. In line with the critical early years developmental period to the point where children are expected to be fully proficient in FMS, the participants must have been aged 2–7 years, with the data ascertained from normally developing children, free of disability or co-ordination difficulties. Data could be collected from children of typical weight status or from overweight or obese children on the condition that there were no related comorbidity indicators. FMS are measured by multiple tools around the world. Therefore, studies that measured FMS using an accepted and validated method, such as the Test of Gross Motor Development, Second/Third Edition (TGMD-2/3), or the Peabody Developmental Motor Scales, Second Edition (PDMS-2), were accepted.

Review articles were not considered for this work. Studies were excluded if the participants were outside of the target range of 2–7 years; the participants were clinically diagnosed with disabilities, morbidities, or co-ordination difficulties; the intervention did not explicitly involve the parental component; quantitative FMS data were not used as an outcome measure; the literature was not published or peer-reviewed.

### 2.3. Search Strategy

**Figure 1:**
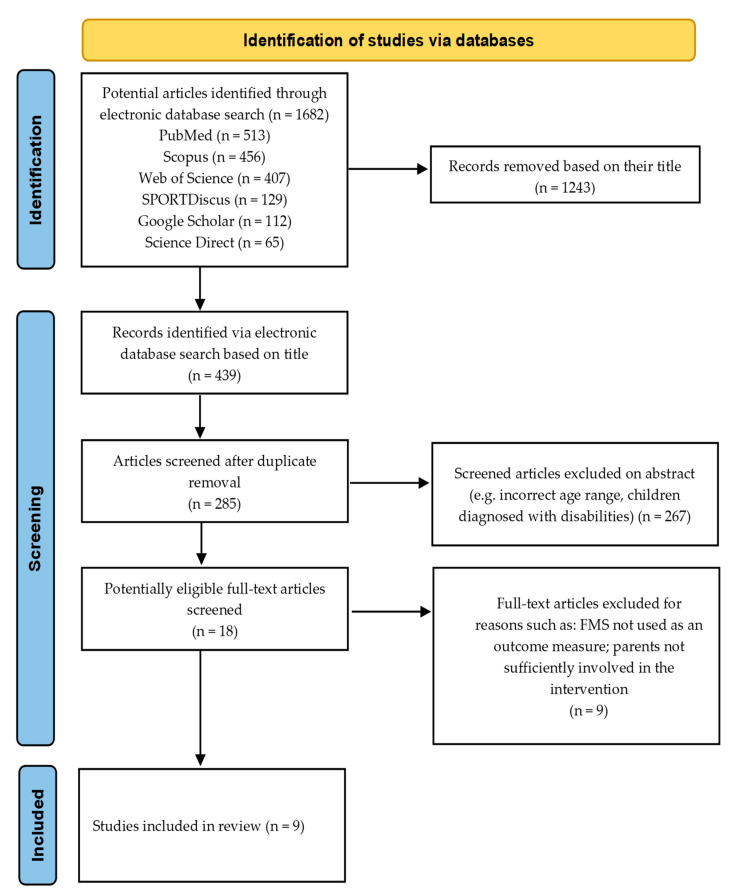
Preferred Reporting Items for Systematic Reviews and Meta-Analyses (PRISMA) flowchart.

A tailored literature search of electronic databases that included Scopus, Web of Science, SPORTDiscus, PubMed, Science Direct, and Google Scholar was carried out up to the 30 November 2022, applying all combinations of the following key words within the titles: fundamental movement skills, fundamental motor skills, motor skills, motor competency, motor proficiency, physical literacy; parent, guardian, mother, father, family, home, community; physical activity, exercise, intervention, programme, assessment, promotion, education; children, early childhood, preschool children, early years. The titles were screened according to the criteria and duplicated papers from separate search engines were subsequently removed. An additional screening of the abstract was undertaken, and in the case of uncertainty as to whether the inclusion criteria had been fulfilled, the article was included in the full text screen. Full-text articles were reviewed for eligibility. The search strategy was completed by the lead researcher (RF) and may be viewed in **(**Figure 1). The original search sample was later shared with the second researcher (CR) to ensure agreement on the inclusion of studies. For studies that were not initially agreed upon, a discussion was held to reach a mutual decision on the inclusion of specific articles. A final search was carried out prior to writing to check for new updates since the initial search.

For all eligible articles that fulfilled the inclusion criteria, the following data were extracted: author(s), year of publication, country of origin, study design (randomised controlled trial, quasi-experimental study), setting, parental involvement, intervention description, sample sizes of the children and of the parents if specified, FMS assessment tool(s) used, and overall findings regarding FMS proficiency.

### 2.4. Study Quality Assessment

The mixed methods appraisal tool (MMAT) [80] was applied to provide a quality score that would indicate the strength of evidence and risk of bias within the studies included in the review. However, this was not used to determine the inclusion or exclusion of the individual studies from the review. Two initial questions were used to screen the studies, followed by an appraisal via five criteria corresponding to their study design category. The category of study included in this review were randomised controlled trials (RCTs), quasi-experimental studies, and an exploratory pilot study. The questions and criteria received “yes”, “no”, or “cannot tell” responses. A response of “yes” would obtain one mark and “no” or “cannot tell” would receive zero marks. Therefore, an article may receive a maximum score of 7.

### 2.5. Analysis

The data were explored via a narrative analysis, adopted because of an absence of heterogeneity of the data with regards to diversity amongst populations, outcome measures, and multiple methods included. This offered a holistic insight into the intricacies of the associated effects and provided objective conclusions for the reasons for these outcomes.

## 3. Results

### 3.1. Study Selection

A total of 1682 articles that included potential duplicates were identified using the key word search across six online search engines. Subsequently, 1243 articles were discarded based on their title, followed by the removal of duplicate articles and the exclusion of a further 267 articles after examination of the abstract. The remaining 18 articles were considered for full-text eligibility and nine articles were included for the final analysis. The common reasons for omission included a lack of or insufficient parental involvement in the PA interventions, the FMS not being used as an outcome measure, unrecognised FMS assessment battery measures, and incorrect age group of the children, as shown in Figure 1.

### 3.2. Origin and Participants

The nine articles included were from several countries of origin. Three articles were from the United States of America (USA), three from Canada, two from Australia, and a single study was based in Finland. The total sample number of children for this review was 743 participants. The average participant number was 83, with a range of 11 to 215 participants. The mean age of the children involved was 4.4 years. The gender of the participants was reported in all nine articles and consisted of 52% boys and 48% girls. Of the nine articles analysed, five articles reported the sample size of the adult participants. Therefore, the total number of known adult participants was 426, with an average participant number of 85, with a range of 11 to 134 participants. Of the five articles, three articles reported the genders of the adults, which comprised 52% males, 46% females, and 2% guardians. A further three articles specified the ages of the adult participants, the average age of which was 37.1 years.

### 3.3. Study Design

Most of the nine articles included were forms of RCTs. Three of the articles were RCTs and an additional two articles were cluster RCTs. A further three articles were of a quasi-experimental design and one article was an exploratory pilot study.

### 3.4. Study Quality Assessment

The MMAT was used to evaluate the overall quality of the nine articles included in this review. Five articles were assessed via the quantitative randomised controlled trial criteria, while four articles were similarly assessed under the quantitative non-randomised studies criteria. According to the MMAT, most of the studies were of excellent quality. Six of the articles met all seven criteria presented by the tool and may be considered highly rated. An additional two articles met six of the MMAT criteria and one study met five criteria. The individual scores of these studies may be viewed in Table 1.

### 3.5. Settings and Parental Involvement

The most common interventional setting of the nine studies analysed was within the childcare setting. In total, six of the interventions were based in childcare or community centres. The parental involvement in these six articles was diverse. Of the six centre-based interventions, four involved joint parent–child participation in sessions delivered by instructors within the centres with additional takeaway programmes and activities to be completed in the home. In a further two studies, the parents did not take part in the on-site sessions but instead received education to deliver a programme to their children at home. The remaining three articles involved interventions exclusively engaging the parents in the home environment. Two of the interventions utilised smartphone apps, with one study focusing on joint parent–child participation in games and one preferring a structured motor skill programme for the parent to deliver to the child. The final study intervened with parent education and counselling to promote positive PA behaviours at home. The durations of the nine interventions ranged from eight weeks to 52 weeks. Descriptions of the interventions may be viewed in Table 2.

### 3.6. Outcome Measures

The outcome measures that consistently came through in the research were the TGMD-2, TGMD-3, and PDSM-2, while the Korperkoordiantiontest für Kinder (KTK) and the Throwing and Catching Ball (TCB) test from the APM inventory manual and test booklet were also observed. Among the nine articles, three articles utilised the TGMD-2. Another three articles reported via an updated version of the TGMD-2, known as the TGMD-3. A further two articles applied the PDSM-2 and one article used both the KTK and TCB tests, which are more commonly used in Finland. All of these protocols are recognised and validated measures of FMS proficiency in children and have been shown to be highly reliable assessments, as specified by the inclusion criteria [84,85,86,87]. Therefore, the articles containing these measures were accepted for this review. Overall, all nine research articles collated for the analysis had employed either full, partial, or adapted versions of the test protocols, which involved the removal of unwanted or unnecessary skills and the supplementation of more relevant skills according to the study outcomes and demographics. However, due to a lack of heterogeneity of these protocols, a meta-analysis could not be conducted.

### 3.7. Overall Findings

The parental interventions were shown to elicit improvements in children’s FMS proficiency in all nine articles analysed. Significant changes occurred in at least one component of the FMS in seven of the nine articles (*p* < 0.05). Two articles reported on FMS development collectively. Bedard et al. (2017) [60] demonstrated significant improvements in gross motor scores (*p* < 0.05) and James et al. (2020) [78] communicated favourable but non-significant improvements in FMS proficiency (*p* = 0.19). A further six articles described findings specifically related to locomotor skills. The locomotor skill proficiency improved significantly in three of the six articles (*p* < 0.01) and non-significantly in two articles (*p* > 0.05), whereas Laukkanen et al. (2015) [83] found no study effect on locomotor skill development (*p* = 0.737). Object control proficiency was reported in eight of the nine articles. Of the eight articles, six interventions exhibited significant changes in the children’s object control (*p* < 0.05), while two interventions showed non-significant effects on object control development (*p* > 0.05). No articles communicated findings specifically related to postural control.

## 4. Discussion

The primary aim of this study was to review the most up to date evidence and current literature that directly involved parents in PA interventions to improve FMS proficiency in 2–7-year-old children. The further aims were to explore interventional setting and methods to establish a recommendation as to which form of parental engagement could be most valuable to motor competence in children. The FMS in children improved in all nine studies included in this review, demonstrating that high parental participation can be of great benefit to children’s FMS and should be encouraged in future interventions. The interventional setting does not appear to be a defining component of FMS outcomes when parents are involved. However, the key methods that can facilitate positive parental influence on children’s FMS are parent–child co-activity, the education and empowerment of parents to deliver FMS support to their children, and the provision of clear FMS guidance for parents to adhere to. An effective method of delivering these components in addition to increasing the accessibility and feasibility of FMS practice in the family home is the use of smartphone apps, which could be integral to future parent-focussed FMS interventions.

This work expands on a similar systematic review published by Stevenson, Wainwright, and Williams (2022) [73], which evaluated motor skill interventions that included direct and indirect parental involvement within the studies. Stevenson and colleagues [73] concluded that direct parent involvement was superior to indirect, and that the active participation of the parent may be the most influential form of parental engagement in relation to motor competence outcomes. However, since these recommendations, the recent emergence of smartphone apps as a vehicle to directly engage parents and children in FMS practice has potentially laid the foundations for future motor skill interventions, which merits further consideration. Therefore, to the best of our knowledge, this review is the first to specifically investigate the efficacy of direct and explicit parent involvement to improve children’s FMS. Furthermore, the evaluation of the interventional settings and methodologies for parent-focused FMS interventions that included the use of smartphone technology is novel at the time of writing.

The FMS ability in children showed improvement in all nine studies in this review, with seven of these studies demonstrating significant changes, indicating that direct parental involvement in FMS interventions can indeed positively influence motor competence outcomes in young children. This is consistent with the findings of a similar review conducted by Stevenson, Wainwright, and Williams (2022) [73] and reinforces the recommendation that parents should be highly involved in interventions targeting the motor skills of children. These findings also align with an earlier study that may have been the first to involve parents in FMS practice and determined that active involvement could improve the FMS ability in children [88]. However, despite the clear historical and current promise of parent-focused interventions, there still remains a severe lack of research into this particular area. Only nine articles met the inclusion criteria for this review, none of which were completed in the UK. This is surprising, since the literature has strongly suggested that parents can be hugely influential on their children’s PA behaviours [61,62,66,67]. Considering that British children continue to underperform in FMS [7,14], the persistent paucity of research with direct parent involvement is concerning and requires urgent research attention in the future.

Studies that involved parents in childcare-based settings with teacher-led instruction were effective at improving the children’s FMS, as were studies that were parent-led and within the home environment. Thus, supporting parents could be crucial for the delivery of clear messaging into the home environment [89]. These outcomes contrast with previous assumptions that professional instruction is the superior method of developing children’s FMS [90]. Therefore, it is reasoned that the interventional setting is a less defining factor than parent involvement. Of greater importance may be the provision of a clear structure and guidance for parents to follow. Certainly, a commonality of the most successful studies in this review is the presence of highly organised activities and session plans to guide home practice. It has been previously considered, however, that session plans are only appropriate if parents have prior familiarisation with the skills and activities [73]. This conflicts with two of the studies in this review that enabled the parental delivery of FMS via smartphone apps without prior parent orientation to the skills [76,77]. The least effective intervention in this review showed a complete absence of relevant FMS direction [83], which perhaps is a further indicator that appropriate support for parents is a necessity. Therefore, it is proposed that structured guidance for parents via session plans is an integral component for FMS delivery to children, and this may be most effective through the use of smartphone apps.

With regards to the prescriptive element of the studies included, improvements in FMS were detected in as few as eight weeks [28,77,82] and as many as 26 weeks [29,81], while significant changes were demonstrated in interventions that employed short practice sessions at high frequencies [76,82] and by those that used longer session lengths with lower frequencies [28,60]. From this evidence, it can be deduced that the prescription of an intervention can be highly variable yet still effective when parents are involved. These findings closely resemble similar research that communicated how a wide range of FMS interventions had yielded improvements irrespective of the recognisable variability in the duration, frequency, and intensity of the prescriptions [71]. However, this was debated by a recent systematic review that proposed that higher frequencies of exercise per week with session durations in excess of 30 min are required to elicit larger effects on FMS [90]. In contrast, it has been argued that this would increase the burden on parents who may respond more preferably to smaller measures due to time constraints, and this could permit similar enhancements to higher-dose prescriptions [60]. This is contradicted, however, by qualitative parental data that suggested that the sessions could feel rushed and overwhelming when the materials are condensed into smaller periods [78]. Nevertheless, it may be postulated that parent-focused interventions improve children’s FMS, irrespective of the duration and frequency of the sessions, although they may remove the necessity for high-dose interventions.

Childcare-based interventions with additional home components were the most common interventional method, which reported positive outcomes overall. These findings are in line with a similar review that concluded that preschool-based programmes were a positive determinant of FMS in young children [91], and both preschool instruction and parent participation may be crucial to continued learning in the home environment [71]. Two studies in this review achieved success by educating parents as a basis for a parent-led delivery of FMS to their children at home, which resulted in significant changes [81,82]. This demonstration of empowerment may substantiate the existing literature, which has advocated for the education of parents to enhance PA and FMS in children [66,67,70]. However, Altunsoz and Goodway (2016) [82] reported low workshop attendance by parents and poor completion of home activity sheets (16.66%), raising questions against parental compliance and the actual impact on FMS, considering the children also received professional in-centre instruction. An explanation for this weak compliance may be because parents are more likely to misplace, forget, or disregard paper handouts [78], and so this may not be a reliable method of parent engagement. In the study by Wasenius et al. (2018) [81], there were less favourable changes in object control compared to Altunsoz and Goodway (2016) [82], possibly as a consequence of a reduced object control emphasis compared to locomotion (33% vs. 50%) to allow for creative play inclusion. Similar interventions with greater object control outcomes have placed more prominence on object control instruction [28,92], and this may be necessary to elicit greater change in such skills.

Another method that resulted in positive findings was parent–child co-activity [28,29,60,78]. The research has ascertained that the FMS of parents may be significantly associated with the motor competency of their children [93]. Hence, the joint participation of parents and children is deemed to be highly influential on the children’s FMS, as it encourages reinforcement and parent role modelling, which enable children to learn through parental example [73]. In this review, Morgan et al. (2022) [28] established that the co-activity of fathers and children could lead to significantly improved object control skills. The fathers’ participation can be construed positively, as they are thought to be more likely to directly support PA compared to mothers [94]. However, the exclusion of mothers, overrepresentation of boys, and omission of locomotor skills as an outcome measure may have created gender bias in favour of object control development, considering males generally favour and outperform girls in object control [5,30]. Furthermore, paternal involvement is often more beneficial to boys and object control than girls and locomotor abilities due to gender-typed behaviour [63]. The findings would, therefore, have been more reliable and representative of the wider population with an equal ratio of females to males and the inclusion of locomotor skills. Despite these limitations, the encouraging findings are in support of parent–child co-activity as an effective method of improving children’s FMS.

One co-activity intervention did not unfold as originally intended [29]. According to the district’s policy, all families that were enrolled were afforded access to the intervention, thereby preventing randomisation. This may have impacted the reliability due to the lack of a control group, although control participants rarely show changes during interventions [95], and so the loss of randomisation may not have been detrimental to the findings. An additional barrier was the severe disruption caused by COVID-19, which prohibited family attendance in school-based sessions for the final three months of the programme and obstructed the immediate post-testing of the children’s FMS. Despite these difficulties, very promising and significant results in terms of both locomotor and object control skills were reported. This may have been because the families still received six school-based sessions before the forced curtailment and had uninterrupted access to online materials at home until the intervention ceased. However, the researchers were unable to track online engagement, and later realised that the rural-residing and socio-economically disadvantaged participants did not all have access to a home computer. The financial constraints of low-income families represent one of many interrelated barriers that prevent FMS development in children living in deprived areas, unless appropriately addressed [38]. Brian and colleagues [29] proposed that mobile applications could be utilised in future work to make the implementation easier and more accessible for these families and remove the burden of attending in-centre sessions. This concept is supported by a recent qualitative study that concluded that the ubiquitous use of mobile devices among families provided a unique and innovative opportunity to reach rural and low-income groups for FMS purposes [96]. Therefore, future research would clearly benefit from the use of smartphone apps to deliver FMS interventions to families of all backgrounds.

Indeed, two of the three studies in this review that exclusively engaged parents in the home environment intervened via smartphone apps, both producing considerable results [76,77]. Mobile devices are now integral to everyday family routines, and parents are generally supportive of their use as a tool for behavioural management, including FMS delivery [96]. Consequently, digital PA interventions are an increasingly appealing method as they may improve the access and practicality for parents and are low in cost compared to in-person interventions [97]. Both digital studies included reported high levels of engagement that may have contributed to the improvement in FMS, possibly because the families found the apps to be fun and engaging, user friendly, and flexible to allow convenient access from the comfort of the home [77]. This view concurs with comparable parental opinion that enjoyment and positive experiences during an intervention are important attributes that engage children and maintain their interest in FMS practice [78]. In light of this, it could be postulated that feasibility, accessibility, practicality, and enjoyment are valuable traits of successful parent-focused FMS interventions. However, in both studies, a limitation was that although the login details could confirm regular use of the app, it was not possible to track participation and fidelity without parental self-reporting, which may have introduced reporting bias. Additionally, the relatively homogeneous sample in the study by Staiano et al. (2022) [76] could have influenced the findings. However, families were not blinded to group allocation by Trost and Brookes (2021) [77], and so behaviour changes could have occurred in the control group, which may have reduced the significance of the locomotor skills. Despite these shortcomings, the promise shown by digital interventions has highlighted the potential for wider distribution and may be integral to future FMS interventions.

The final home-based intervention involved parent counselling and PA promotion [83], which was the least effective method of parent engagement in this review. Almost significant improvements were seen in object control skills and there was no study effect for locomotor skills. One reason for the lesser findings may have been due to the primary focus of the study being to increase MVPA rather than FMS development, and so no structure or guidance was provided for activity choice or practice. The intervention considered the promotion of physical activity in natural environments around the home to be influential on the motor competence of children. Certainly, the view that nature-based play can enhance FMS does exist within the literature [98,99]. However, the belief that children would intrinsically develop attributes related to FMS through the promotion of physical activity in the natural environment meant that no structured FMS support was served to the parents. Moreover, after the initial counselling, the parents were only contacted indirectly by email or telephone after two months and five months, during a year-long intervention, which may have been insufficient to reinforce the initial messaging. As the need for structured guidance for families to improve FMS has been identified earlier in this review [80,81,83], this would offer a reasonable explanation as to why counselling and PA promotion may not have provided sufficient guidance for parents to effectively change children’s FMS. Furthermore, a long study period of 12 months with minimal contact may not have been stimulating enough to sustain engagement. However, the harsh weather conditions in Finland may have caused a seasonal impact, as when the influence of the season was considered, a greater locomotor skill change was revealed. Therefore, family-based PA counselling may still play a role in motor competence development.

Overall, there were several aspects that rendered the interpretation of interventional effectiveness in this review challenging. Small sample sizes were a common theme within the studies. Six studies had fewer than 100 participants, potentially leaving them insufficiently representative of the wider population and underpowered to detect changes. Nevertheless, the collective sample size of this review was a sizeable 743 children, which allowed the overall results to be used as a collective representation of 2–7-year-old children. An additional challenge was the inconsistent reporting of the parent sample and gender information, which could have revealed useful trends if made available. With little parent information presented, it is difficult to determine if any bias or gender-typed behaviour may have occurred within the studies. There was further unpredictability regarding the choice of FMS assessment tool depending on the country of origin. The variability in motor competence examinations is an ongoing issue that makes comparisons of FMS outcomes challenging within the field and may continue to be problematic until a general consensus is reached amongst researchers regarding FMS the measurements and methodology [12]. A recommendation by Robinson and colleagues [12] is the combined use of assessments such as the TGMD-2 and the KTK, which would create more a holistic and comparable FMS assessment between countries. Conversely, this approach may be arduous and unrealistic for young participants and may negatively impact their engagement.

An important observation was that FMS were not the primary outcome in every study included, leading to partial or adapted test protocols according to differing study aims. For instance, Morgan et al. (2022) [28] prioritised PA levels in children as a primary outcome, and so the object control measurement was deemed sufficient for the FMS assessment as a secondary outcome. Locomotor skills were similarly omitted by Altunsoz and Goodway (2016) [82], as they intended to further examine significant improvements in object control demonstrated by an earlier intervention by Hamilton et al. (1999) [88]. Although Altunsoz and colleagues [82] reported significant changes in object control, the exclusion of a locomotor assessment prevented a complete assessment of the parental influence on FMS in this instance. Adaptations were also carried out by Bedard et al. (2017) [60] and James et al. (2020) [78] to include reading literacy and social emotional learning in addition to FMS within their respective programmes, and this led to reduced FMS practice and may have lessened the FMS changes. However, despite the possible limitations related to partial or adapted testing, positive outcomes were still obtained from these studies.

### 4.1. Strengths and Limitations

The current review successfully collected and holistically considered all available and up to date research relating to meaningful parental involvement in children’s FMS and was able to form important recommendations in an area that is currently underrepresented. Moreover, this review was the first to evaluate the involvement of smartphone apps in children’s FMS development. Virtual learning is particularly important, not only for the dissemination of FMS guidance to families of all backgrounds [29,95,96] but to help negate the potential decline in children’s FMS due to unforeseen future events, as demonstrated by the lockdowns of the COVID-19 pandemic [29,100]. The other strengths are the robustness of the methodology, the inclusion criteria, and the justification of what was quantified as “direct and explicit” parent involvement. This ensured that lesser, indirect parent contributions that may have convoluted the analyses were omitted and the most relevant research was utilised to form recommendations.

As with all studies there were limitations to this review. Although the definition of direct parent involvement was justified, this interpretation was still subjective in nature and may be interpreted differently by other researchers. This could have resulted in relevant articles being overlooked. There was an absence of heterogeneity within the data, which prevented a meta-analysis. Due to the adoption of a narrative analysis, the interpretation of trends and findings should, therefore, be treated with caution. Another limitation may have been the exclusion of older children from this review. During the tailored literature search, certain articles were discarded after failing to meet the inclusion criteria due to the incorrect age of the children. However, these articles involved direct engagement of the parent and demonstrated positive changes in the children’s FMS [56,74] that may have added key summary knowledge of how parent-focused FMS interventions can influence adolescent children. Thus, older children could be considered in future work. This review could also have been extended to investigate the retention of skills following parent interventions, as there is evidence that the enhancements may endure [28,29]. Comparatively, skills are not always retained through teacher-led school-based programmes [101]. However, considering the lack of research into retention, the risk of bias within the school setting [36], and the scarcity of interventions with parent involvement in general, it may not have been feasible to draw meaningful comparisons at the time of writing.

### 4.2. Practical Implications

Considering the current inadequacy of children’s FMS, PA engagement, and parental research focus, both domestically and internationally, it may be of benefit to share findings from this study through appropriate professional networks to provide others with the opportunity to begin to reflect on this research. For example, contact will be made with Active Derbyshire and the Active Notts Physical Activity Teams, who have a shared vision to empower both people and families within the East Midlands region to become more physically active via the Making Our Move campaign [102]. Active Derbyshire and Active Notts are members of a collective network of Active Partnerships that are supported by Sport England and extend nationally through many organisations across England and may be ideal to facilitate the propagation of ideas [102]. Equally, it may be important to connect with associations that embrace the importance of parenting and parental influence, such as The First 1000 Days Yorkshire Project, to extend learning to parents with young children [103]. The project is a major evidence-based participatory initiative aimed at addressing health inequalities by promoting, enabling, and modelling healthy behaviours and relationships in the first 1000 days of a child’s life to improve their health and wellbeing in adulthood [103], and may be enhanced by educating parents on the importance of children’s motor competence. Further, this paper highlights key research gaps and questions for future investigations on FMS in children that could be presented to the International Motor Competence Network (IMCNetwork) [104] and the International Motor Development Research Consortium (I-MDRC) [105], which are collaborations of academics and researchers who wish to promote and translate global knowledge regarding motor development research. Furthermore, the education and empowerment of parents to support the self-delivery of FMS to their children within their home environment is essential, with the emergence of smartphone apps potentially increasing the feasibility, accessibility, and enjoyment of FMS practice for parents and children.

## 5. Conclusions

Direct parent involvement can effectively elicit improvements in FMS in 2–7-year-old children and further research is clearly warranted. The findings from this review indicate that both the childcare setting and the home environment are equally appropriate for parents to engage with FMS practice and improve the motor competency of their children. The important components that contribute to the success of these interventions appear to be the education and empowerment of the parent to support the self-delivery of FMS and the co-participation of parents and children, which encourages role modelling and enables children to learn by their parents’ example. The provision of a clear structure and support through session plans and physical activity guidance for parents to follow are also important features that may allow the transfer of messaging into the home environment for continued learning. The recent emergence of smartphone apps has potentially removed the burden and cost of specialist-led instruction and may increase the feasibility, accessibility, and enjoyment of FMS practice for parents and children. To our knowledge, this is the first study to review the use of smartphone technology as a means to improve FMS in children exclusively within the family home; therefore, it adds important insight into an area that may be integral to the future effectiveness of parent interventions.

## Figures and Tables

**Table 1:** MMAT quality assessment outcomes.

Author and Year of Publication	MMAT Score
Trost and Brookes, 2021 [77]	7
Staiano et al., 2022 [76]	7
Wasenius et al., 2018 [81]	7
Morgan et al., 2022 [28]	7
James et al., 2020 [78]	6
Altunsoz and Goodway, 2016 [82]	7
Brian et al., 2022 [29]	5
Laukkanen et al., 2015 [83]	6
Bedard et al., 2017 [60]	7

**Table 2:** Descriptive results of PA interventions with direct parental involvement and the influence on children’s FMS proficiency.

Author & Country	Study Design	Setting	Parent Component Method and Intervention Description	Duration (Weeks)	Sample	Mean Child Age (Years)	FMS Assessment Tool(s)	Overall Findings
Trost and Brookes (2021) [77]. Australia	RCT	Home	**Smartphone app for joint parent–child participation:** A digital games library of physical literacy skills to promote parent–child participation in 60 min of daily MVPA and to enhance FMS.	8	34 parent–child dyads: 17 boys 17 girls	5.3 ± 1.2	**TGMD-2**: 6 LOCO skills (run, gallop, hop, leap, jump, slide); 6 OC skills (strike, dribble, catch, kick, throw, roll).	(1) Non-significant between-group improvements in LOCO skills (*p* = 0.085). (2) Significant between-group improvements in OC skills (*p* = 0.003).
Staiano et al. (2022) [76]. USA	RCT	Home	**Smartphone app for parent home delivery:** 12 h (12 min per day, 5 × per week) of structured motor skills delivered by parents at home.	12	72 children: 31 boys 41 girls	4.0 ± 0.8	**TGMD-3:** 6 LOCO skills (run, gallop, skip, hop, jump, slide); 7 OC skills (two-hand strike, one-hand strike, dribble, catch, kick, throw, roll).	(1) Significant between-group improvements in LOCO skills (*p* < 0.01). (2) Significant between-group improvements in OC skills (*p* < 0.01).
Laukkanen et al. (2015) [83]. Finland	Cluster RCT	Home	**Parent education and counselling:** Parents received one lecture followed by face-to-face counselling and goal-setting to promote PA. Telephone discussions for reinforcement held at 2 months and 5 months.	52	91 children: 42 boys 49 girls 122 parents 52 males 70 females	6.2 ± 1.1	**KTK:** Walking backwards, hopping for height, jumping sideways, moving sideways. **TCB test** from APM Inventory.	(1) No study effect for LOCO skills (*p* = 0.737). (2) Non-significant between-group improvements in OC skills after 6 months (*p* = 0.051) but did not differ at 12 months (*p* = 0.984).
Wasenius et al. (2018) [81]. Canada	Cluster RCT	Hybrid: Children’s Centre and Home	**Online parent education and paper materials for parent home delivery:** Childcare providers received 2 × 3 h workshops and bi-monthly booster sessions to deliver a structured activity programme to the children within the centre. Parents received two online webinars of similar content to the childcare providers, an ABC manual to follow with the children at home, and bi-weekly postcards to encourage PA at home and in the community.	26	215 children: 117 boys 98 girls	3.6 ± 0.5	**TGMD-2:** 6 LOCO skills (run, gallop, hop, leap, jump, slide); 6 OC skills (strike, dribble, catch, kick, throw, roll).	(1) Significant between-group improvements in LOCO skills (*p* < 0.001). (2) Significant within-group improvements for OC skills (*p* < 0.001) but no significant between-group changes (*p* > 0.05).
Brian et al. (2022) [29]. USA	Quasi-experimental Study	Hybrid: Childcare Centre and Home	**Parent education & joint parent–child participation:** 1 × 60-min centre-based session per month (30 min parent education, 30 min parent–child participation. Parents provided with online materials to encourage FMS and PA engagement at home.	26	104 children: 53 boys 51 girls 134 parents: 20 males 106 females 8 guardians	5.1 ± 0.5	**TGMD-3**: 6 LOCO skills (run, jump, hop, gallop, skip, slide); 7 OC skills (dribble, strike with bat, strike with paddle, underarm throw, overarm throw, kick, catch).	(1) Significant improvements in LOCO skills (*p* = 0.008) with non-significant retention at 12 months (*p* = 0.24). (2) Significant improvements in OC skills (*p* < 0.001) that were significantly retained at 12 months (*p* = 0.018).
Morgan et al. (2022) [28]. Australia	RCT	Hybrid: Childcare Centre and Home	**Parent education & joint parent–child participation:** 2 × 2-h fathers-only face-to-face workshops. 8 × 75-min father-child practical sessions delivered in centre. Activity handbook for fathers and children to complete at home.	8	125 father-child dyads: 76 boys 49 girls	3.9 ± 0.5	**TGMD-3:** 5 OC skills (two-hand strike, one-hand strike, dribble, overarm throw, underarm throw).	(1) Significant between-group improvements in OC skills at 10 weeks (*p* < 0.001) and at 9 months (*p* = 0.011).
Altunsoz and Goodway (2016) [82]. USA	Quasi-experimental Study	Hybrid: Childcare Centre and Home	**Parent education for home delivery:** 16 × 30-min OC skill development sessions delivered in centre by a trained motor skill instructor. Parents received a 1.5-h workshop to prepare to deliver 24 × 10–15-min sessions to their children at home. A parent–child motor activity calendar was provided to be followed at home.	8	72 children: 36 boys 36 girls	4.0 ± 0.6	**TGMD-2**: 6 OC skills (strike, dribble, catch, kick, throw, roll).	(1) Significant between-group improvements in OC skills (*p* < 0.001).
James et al. (2020) [78]. Canada	Exploratory Pilot Study	Hybrid: Community Centre and Home	**Joint parent–child participation:** 1 × 60-min session per week for 10 weeks delivered by master’s students and undergraduate volunteers, involving direct FMS instruction, free play, and social emotional learning. Parents and caregivers actively participated and were provided with a take home guide to practice activities at home.	10	11 parent–child dyads: 7 boys 4 girls	4.2 ± 0.7	**PDMS-2:** Stationary performance (30 items), locomotion (89 items), object manipulation (24 items).	(1) Non-significant improvements in FMS (*p* = 0.191).
Bedard et al. (2017) [60]. Canada	Quasi-experimental Study	Hybrid: Community Centre and Home	**Joint parent–child participation:** 1 × 60-min session per week for 10 weeks delivered by graduate students, involving direct FMS instruction, free play, and storybook reading. Parents and caregivers actively participated in the sessions. Weekly handouts were provided with activities to be practiced at home.	10	19 children: 10 boys 9 girls	3.7	**PDMS-2:** Stationary performance (30 items), locomotion (89 items), object manipulation (24 items).	(1) Non-significant between-group improvements in LOCO skills (*p* = 0.14). (2) Significant between-group improvements in OC skills (*p* < 0.05). (3) Significant between-group improvements in gross motor score (*p* < 0.05).

RCT: randomised controlled trial; PA: physical activity; TGMD: Test of Gross Motor Development; PDMS: Peabody Developmental Motor Scale; KTK: Korperkoordiantiontest for Kinder; TCB: throwing and catching ball; APM: Assessment of Perceptual and Fundamental Motor Skills; LOCO: locomotor; OC: object control; FMS: fundamental movement skills; MVPA: moderate to vigorous physical activity; ABC: Activity Begins in Childhood; &: and. For the purpose of the table, the author has streamlined the age of the participants into years and the duration of the interventions into weeks.

## Data Availability

No new data were created or analysed in this study. Data sharing is not applicable to this article.

## References

[1] Seefeldt V. (1980). Developmental motor patterns: Implications for elementary school physical education. Psychol. Mot. Behav. Sport.

[2] Wick K., Leeger-Aschmann C.S., Monn N.D., Radtke T., Ott L.V., Rebholz C.E., Cruz S., Gerber N., Schmutz E.A., Puder J.J. (2017). Interventions to promote fundamental movement skills in childcare and kindergarten: A systematic review and meta-analysis. Sports Med..

[3] Rudd J.R., Barnett L.M., Butson M.L., Farrow D., Berry J., Polman R.C. (2015). Fundamental movement skills are more than run, throw and catch: The role of stability skills. PLoS ONE.

[4] Foulkes J.D., Knowles Z., Fairclough S.J., Stratton G., O’Dwyer M., Ridgers N.D., Foweather L. (2015). Fundamental movement skills of preschool children in Northwest England. Percept. Mot. Ski..

[5] Morley D., Till K., Ogilvie P., Turner G. (2015). Influences of gender and socioeconomic status on the motor proficiency of children in the UK. Hum. Mov. Sci..

[6] Stodden D.F., Goodway J.D., Langendorfer S.J., Roberton M.A., Rudisill M.E., Garcia C., Garcia L.E. (2008). A Developmental Perspective on the Role of Motor Skill Competence in Physical Activity: An Emergent Relationship. Quest.

[7] Lawson C., Eyre E.L., Tallis J., Duncan M.J. (2021). Fundamental movement skill proficiency among British primary school children: Analysis at a behavioral component level. Percept. Mot. Ski..

[8] Ali A., McLachlan C., Mugridge O., McLaughlin T., Conlon C., Clarke L. (2021). The effect of a 10-week physical activity programme on fundamental movement skills in 3–4-year-old children within early childhood education centres. Children.

[9] Cohen K.E., Morgan P.J., Plotnikoff R.C., Callister R., Lubans D.R. (2014). Fundamental movement skills and physical activity among children living in low-income communities: A cross-sectional study. Int. J. Behav. Nutr. Phys. Act..

[10] Holfelder B., Schott N. (2014). Relationship of fundamental movement skills and physical activity in children and adolescents: A systematic review. Psychol. Sport Exerc..

[11] Vandorpe B., Vandendriessche J., Vaeyens R., Pion J., Matthys S., Lefevre J., Philippaerts R., Lenoir M. (2012). Relationship between sports participation and the level of motor coordination in childhood: A longitudinal approach. J. Sci. Med. Sport.

[12] Robinson L.E., Stodden D.F., Barnett L.M., Lopes V.P., Logan S.W., Rodrigues L.P., D’Hondt E. (2015). Motor competence and its effect on positive developmental trajectories of health. Sports Med..

[13] Bolger L.E., Bolger L.A., O’Neill C., Coughlan E., O’Brien W., Lacey S., Burns C., Bardid F. (2021). Global levels of fundamental motor skills in children: A systematic review. J. Sports Sci..

[14] Duncan M.J., Roscoe C.M., Noon M., Clark C.C., O’Brien W., Eyre E.L. (2020). Run, jump, throw and catch: How proficient are children attending English schools at the fundamental motor skills identified as key within the school curriculum?. Eur. Phys. Educ. Rev..

[15] O’Brien W., Belton S., Issartel J. (2016). Fundamental movement skill proficiency amongst adolescent youth. Phys. Educ. Sport Pedagog..

[16] Dobell A., Pringle A., Faghy M.A., Roscoe C.M. (2020). Fundamental Movement Skills and Accelerometer-Measured Physical Activity Levels during Early Childhood: A Systematic Review. Children.

[17] GOV.UK (2019). Physical Activity Guidelines: Early Years (Under 5s). www.gov.uk.

[18] World Health Organisation (2020). WHO Guidelines on Physical Activity and Sedentary Behaviour. https://www.who.int/publications/i/item/9789240015128.

[19] Hallal P.C., Andersen L.B., Bull F.C., Guthold R., Haskell W., Ekelund U. (2012). Lancet Physical Activity Series Working Group. Global physical activity levels: Surveillance progress, pitfalls, and prospects. Lancet.

[20] Hardy L.L., Barnett L., Espinel P., Okely A.D. (2013). Thirteen-year trends in child and adolescent fundamental movement skills: 1997–2010. Med. Sci. Sports Exerc..

[21] Centers for Disease Control and Prevention (2022). Physical Activity Facts | Healthy Schools | CDC. https://www.cdc.gov/healthyschools/physicalactivity/facts.htm.

[22] Public Health England (2019). Physical Activity Helps Children to Deal with Life’s Challenges. www.gov.uk.

[23] Tinner L., Kipping R., White J., Jago R., Metcalfe C., Hollingworth W. (2019). Cross-sectional analysis of physical activity in 2–4-year-olds in England with paediatric quality of life and family expenditure on physical activity. BMC Public Health.

[24] De Meester A., Stodden D., Goodway J., True L., Brian A., Ferkel R., Haerens L. (2018). Identifying a motor proficiency barrier for meeting physical activity guidelines in children. J. Sci. Med. Sport.

[25] Cliff D.P., Okely A.D., Morgan P.J., Jones R.A., Steele J.R., Baur L.A. (2012). Proficiency deficiency: Mastery of fundamental movement skills and skill components in overweight and obese children. Obesity.

[26] Han A., Fu A., Cobley S., Sanders R.H. (2018). Effectiveness of exercise intervention on improving fundamental movement skills and motor coordination in overweight/obese children and adolescents: A systematic review. J. Sci. Med. Sport.

[27] Siahkouhian M., Mahmoodi H., Salehi M. (2011). Relationship between fundamental movement skills and body mass index in 7-to-8 year-old children. World Appl. Sci. J..

[28] Morgan P.J., Grounds J.A., Ashton L.M., Collins C.E., Barnes A.T., Pollock E.R., Kennedy S.L., Rayward A.T., Saunders K.L., Drew R.J. (2022). Impact of the ‘Healthy Youngsters, Healthy Dads’ program on physical activity and other health behaviours: A randomised controlled trial involving fathers and their preschool-aged children. BMC Public Health.

[29] Brian A., Taunton Miedema S., Starrett A., Griffin S., Stribing A., Miedema B., Walker M., Casner C., Wainwright N., Wadsworth D. (2022). SKIPping with PALS: Exploring Parental Engagement in a Motor Intervention for Their Preschool Children. Res. Q. Exerc. Sport.

[30] Bryant E.S., Duncan M.J., Birch S.L. (2014). Fundamental movement skills and weight status in British primary school children. Eur. J. Sport Sci..

[31] Kokštejn J., Musálek M., Tufano J.J. (2017). Are sex differences in fundamental motor skills uniform throughout the entire preschool period?. PLoS ONE.

[32] Hesketh K.R., Griffin S.J., van Sluijs E.M. (2015). UK Preschool-aged children’s physical activity levels in childcare and at home: A cross-sectional exploration. Int. J. Behav. Nutr. Phys. Act..

[33] Lai S.K., Costigan S.A., Morgan P.J., Lubans D.R., Stodden D.F., Salmon J., Barnett L.M. (2014). Do school-based interventions focusing on physical activity, fitness, or fundamental movement skill competency produce a sustained impact in these outcomes in children and adolescents? A systematic review of follow-up studies. Sports Med..

[34] Olesen L.G., Kristensen P.L., Ried-Larsen M., Grøntved A., Froberg K. (2014). Physical activity and motor skills in children attending 43 preschools: A cross-sectional study. BMC Pediatr..

[35] Hardy L.L., King L., Farrell L., Macniven R., Howlett S. (2010). Fundamental movement skills among Australian preschool children. J. Sci. Med. Sport.

[36] Zheng Y., Ye W., Korivi M., Liu Y., Hong F. (2022). Gender Differences in Fundamental Motor Skills Proficiency in Children Aged 3–6 Years: A Systematic Review and Meta-Analysis. Int. J. Environ. Res. Public Health.

[37] Hardy L.L., Reinten-Reynolds T., Espinel P., Zask A., Okely A.D. (2012). Prevalence and correlates of low fundamental movement skill competency in children. Pediatrics.

[38] Eyre E.L., Adeyemi L.J., Cook K., Noon M., Tallis J., Duncan M. (2022). Barriers and Facilitators to Physical Activity and FMS in Children Living in Deprived Areas in the UK: Qualitative Study. Int. J. Environ. Res. Public Health.

[39] Eyre E.L., Walker L.J., Duncan M.J. (2018). Fundamental movement skills of children living in England: The role of ethnicity and native English language. Percept. Mot. Ski..

[40] Webster E.K., Martin C.K., Staiano A.E. (2019). Fundamental motor skills, screen-time, and physical activity in preschoolers. J. Sport Health Sci..

[41] Spessato B.C., Gabbard C., Valentini N., Rudisill M. (2013). Gender differences in Brazilian children’s fundamental movement skill performance. Early Child Dev. Care.

[42] Capio C.M., Poolton J.M., Sit C.H.P., Holmstrom M., Masters R.S.W. (2013). Reducing errors benefits the field-based learning of a fundamental movement skill in children. Scand. J. Med. Sci. Sports.

[43] Eddy L., Hill L.J., Mon-Williams M., Preston N., Daly-Smith A., Medd G., Bingham D.D. (2021). Fundamental movement skills and their assessment in primary schools from the perspective of teachers. Meas. Phys. Educ. Exerc. Sci..

[44] Dobell A., Pringle A., Faghy M.A., Roscoe C.M. (2021). Educators Perspectives on the Value of Physical Education, Physical Activity and Fundamental Movement Skills for Early Years Foundation Stage Children in England. Children.

[45] Chan C.H., Ha A.S., Ng J.Y., Lubans D.R. (2019). The A+ FMS cluster randomized controlled trial: An assessment-based intervention on fundamental movement skills and psychosocial outcomes in primary schoolchildren. J. Sci. Med. Sport.

[46] Grainger F., Innerd A., Graham M., Wright M. (2020). Integrated strength and fundamental movement skill training in children: A pilot study. Children.

[47] Faigenbaum A.D., Myer G.D. (2012). Exercise deficit disorder in youth: Play now or pay later. Curr. Sports Med. Rep..

[48] O’Sullivan C.Ó., Parker M., Comyns T., Ralph A. (2020). Enhancing Fundamental Movement Skills: Understanding Student Voices. J. Teach. Phys. Educ..

[49] Pot N., van Hilvoorde I., Afonso J., Koekoek J., Almond L. (2017). Meaningful movement behaviour involves more than the learning of fundamental movement skills. Int. Sports Stud..

[50] Cohen K.E., Morgan P.J., Plotnikoff R.C., Barnett L.M., Lubans D.R. (2015). Improvements in fundamental movement skill competency mediate the effect of the SCORES intervention on physical activity and cardiorespiratory fitness in children. J. Sports Sci..

[51] Roscoe C.M., James R.S., Duncan M.J. (2017). Preschool staff and parents’ perceptions of preschool children’s physical activity and fundamental movement skills from an area of high deprivation: A qualitative study. Qual. Res. Sport Exerc. Health.

[52] Coleman B., Dyment J.E. (2013). Factors that limit and enable preschool-aged children’s physical activity on child care centre playgrounds. J. Early Child. Res..

[53] Adamo K.B., Wilson S., Harvey A.L., Grattan K.P., Naylor P.J., Temple V.A., Goldfield G.S. (2016). Does intervening in childcare settings impact fundamental movement skill development. Med. Sci. Sports Exerc..

[54] Leis A., Ward S., Vatanparast H., Humbert M.L., Chow A.F., Muhajarine N., Engler-Stringer R., Bélanger M. (2020). Effectiveness of the Healthy Start-Départ Santé approach on physical activity, healthy eating and fundamental movement skills of preschoolers attending childcare centres: A randomized controlled trial. BMC Public Health.

[55] Tugault-Lafleur C.N., Naylor P.J., Carson V., Faulkner G., Lau E.Y., Wolfenden L., Mâsse L.C. (2022). Does an active play standard change childcare physical activity and healthy eating policies? A natural policy experiment. BMC Public Health.

[56] Ha A.S., Lonsdale C., Lubans D.R., Ng F.F., Ng J.Y. (2021). Improving children’s fundamental movement skills through a family-based physical activity program: Results from the “Active 1+ FUN” randomized controlled trial. Int. J. Behav. Nutr. Phys. Act..

[57] Johnstone A., Hughes A.R., Martin A., Reilly J.J. (2018). Utilising active play interventions to promote physical activity and improve fundamental movement skills in children: A systematic review and meta-analysis. BMC Public Health.

[58] Lane C., Naylor P.J., Predy M., Kurtzhals M., Rhodes R.E., Morton K., Hunter S., Carson V. (2022). Exploring a parent-focused physical literacy intervention for early childhood: A pragmatic controlled trial of the PLAYshop. BMC Public Health.

[59] Roscoe C.M., James R.S., Duncan M.J. (2019). Accelerometer-based physical activity levels differ between week and weekend days in British preschool children. J. Funct. Morphol. Kinesiol..

[60] Bedard C., Bremer E., Campbell W., Cairney J. (2017). A quasi-experimental study of a movement and preliteracy program for 3-and 4-year-old children. Front. Pediatr..

[61] Barnett L.M., Hnatiuk J.A., Salmon J., Hesketh K.D. (2019). Modifiable factors which predict children’s gross motor competence: A prospective cohort study. Int. J. Behav. Nutr. Phys. Act..

[62] Horodyska K., Boberska M., Kruk M., Szczuka Z., Wiggers J., Wolfenden L., Scholz U., Radtke T., Luszczynska A. (2019). Perceptions of physical activity promotion, transportation support, physical activity, and body mass: An insight into parent-child dyadic processes. Int. J. Behav. Med..

[63] Cools W., De Martelaer K., Samaey C., Andries C. (2011). Fundamental movement skill performance of preschool children in relation to family context. J. Sports Sci..

[64] Brandelli Y.N., Stone M., Westheuser V., Huber A.M., Joshi N., Reid A., Stringer E., Stevens D. (2022). Parent Risk Perceptions, Physical Literacy, and Fundamental Movement Skills in Children with Juvenile Idiopathic Arthritis. Pediatr. Phys. Ther..

[65] Corder K., Crespo N.C., van Sluijs E.M., Lopez N.V., Elder J.P. (2012). Parent awareness of young children’s physical activity. Prev. Med..

[66] Liong G.H., Ridgers N.D., Barnett L.M. (2015). Associations between skill perceptions and young children’s actual fundamental movement skills. Percept. Mot. Ski..

[67] Bentley G.F., Goodred J.K., Jago R., Sebire S.J., Lucas P.J., Fox K.R., Stewart-Brown S., Turner K.M. (2012). Parents’ views on child physical activity and their implications for physical activity parenting interventions: A qualitative study. BMC Pediatr..

[68] Kesten J.M., Jago R., Sebire S.J., Edwards M.J., Pool L., Zahra J., Thompson J.L. (2015). Understanding the accuracy of parental perceptions of child physical activity: A mixed methods analysis. J. Phys. Act. Health.

[69] Agard B., Zeng N., McCloskey M.L., Johnson S.L., Bellows L.L. (2021). Moving together: Understanding parent perceptions related to physical activity and motor skill development in preschool children. Int. J. Environ. Res. Public Health.

[70] Lijuan W., Jiancui S., Suzhe Z. (2017). Parental influence on the physical activity of Chinese children: Do gender differences occur?. Eur. Phys. Educ. Rev..

[71] Tompsett C., Sanders R., Taylor C., Cobley S. (2017). Pedagogical approaches to and effects of fundamental movement skill interventions on health outcomes: A systematic review. Sports Med..

[72] Lane C., Carson V., Morton K., Reno K., Wright C., Predy M., Naylor P.J. (2021). A real-world feasibility study of the PLAYshop: A brief intervention to facilitate parent engagement in developing their child’s physical literacy. Pilot Feasibility Stud..

[73] Stevenson A., Wainwright N., Williams A. (2022). Interventions targeting motor skills in pre-school-aged children with direct or indirect parent engagement: A systematic review and narrative synthesis. Education.

[74] Morgan P.J., Young M.D., Barnes A.T., Eather N., Pollock E.R., Lubans D.R. (2019). Engaging fathers to increase physical activity in girls: The “dads and daughters exercising and empowered” (DADEE) randomized controlled trial. Ann. Behav. Med..

[75] Webster E.K., Kracht C.L., Newton R.L., Beyl R.A., Staiano A.E. (2020). Intervention to Improve Preschool Children’s Fundamental Motor Skills: Protocol for a Parent-Focused, Mobile App–Based Comparative Effectiveness Trial. JMIR Res. Protoc..

[76] Staiano A.E., Newton R.L., Beyl R.A., Kracht C.L., Hendrick C.A., Viverito M., Webster E.K. (2022). mHealth Intervention for Motor Skills: A Randomized Controlled Trial. Pediatrics.

[77] Trost S.G., Brookes D.S. (2021). Effectiveness of a novel digital application to promote fundamental movement skills in 3-to 6-year-old children: A randomized controlled trial. J. Sports Sci..

[78] James M.E., Bedard C., Bremer E., Cairney J. (2020). The acceptability and feasibility of a preschool intervention targeting motor, social, and emotional development. Front. Pediatr..

[79] Veldman S.L., Jones R.A., Okely A.D. (2016). Efficacy of gross motor skill interventions in young children: An updated systematic review. BMJ Open Sport Exerc. Med..

[80] Hong Q.N., Fàbregues S., Bartlett G., Boardman F., Cargo M., Dagenais P., Gagnon M.P., Griffiths F., Nicolau B., O’Cathain A. (2018). The Mixed Methods Appraisal Tool (MMAT) version 2018 for information professionals and researchers. Educ. Inf..

[81] Wasenius N.S., Grattan K.P., Harvey A.L., Naylor P.J., Goldfield G.S., Adamo K.B. (2018). The effect of a physical activity intervention on preschoolers’ fundamental motor skills—A cluster RCT. J. Sci. Med. Sport.

[82] Altunsöz I.H., Goodway J.D. (2016). Skiping to motor competence: The influence of project successful kinesthetic instruction for preschoolers on motor competence of disadvantaged preschoolers. Phys. Educ. Sport Pedagog..

[83] Laukkanen A., Pesola A.J., Heikkinen R., Sääkslahti A.K., Finni T. (2015). Family-based cluster randomized controlled trial enhancing physical activity and motor competence in 4–7-year-old children. PLoS ONE.

[84] Iivonen S., Sääkslahti A., Laukkanen A. (2016). A review of studies using the Körperkoordinationstest für Kinder (KTK). Eur. J. Adapt. Phys. Act..

[85] Numminen P. (1995). APM inventory: Manual and test booklet for assessing pre-school children’s perceptual and basic motor skills. Liikunnan Kansanterveyden Julk..

[86] Rey E., Carballo-Fazanes A., Varela-Casal C., Abelairas-Gómez C., ALFA-MOV Project Collaborators (2020). Reliability of the test of gross motor development: A systematic review. PLoS ONE.

[87] Zanella L.W., Valentini N.C., Copetti F., Nobre G.C. (2021). Peabody Developmental Motor Scales-(PDMS-2): Reliability, content and construct validity evidence for Brazilian children. Res. Dev. Disabil..

[88] Hamilton M., Goodway J., Haubenstricker J. (1999). Parent-assisted instruction in a motor skill program for at-risk preschool children. Adapt. Phys. Act. Q..

[89] Riethmuller A.M., Jones R.A., Okely A.D. (2009). Efficacy of interventions to improve motor development in young children: A systematic review. Pediatrics.

[90] Van Capelle A., Broderick C.R., van Doorn N., Ward R.E., Parmenter B.J. (2017). Interventions to improve fundamental motor skills in pre-school aged children: A systematic review and meta-analysis. J. Sci. Med. Sport.

[91] Livonen S., Sääkslahti A.K. (2014). Preschool children’s fundamental motor skills: A review of significant determinants. Early Child Dev. Care.

[92] Donath L., Faude O., Hagmann S., Roth R., Zahner L. (2015). Fundamental movement skills in preschoolers: A randomized controlled trial targeting object control proficiency. Child Care Health Dev..

[93] Scott-Andrews K.Q., Hasson R.E., Miller A.L., Templin T.J., Robinson L.E. (2022). Associations Between Physical Activity and Gross Motor Skills in Parent–Child Dyads. J. Mot. Learn. Dev..

[94] Laukkanen A., Niemistö D., Finni T., Cantell M., Korhonen E., Sääkslahti A. (2018). Correlates of physical activity parenting: The Skilled Kids study. Scand. J. Med. Sci. Sports.

[95] Logan S.W., Robinson L.E., Wilson A.E., Lucas W.A. (2012). Getting the fundamentals of movement: A meta-analysis of the effectiveness of motor skill interventions in children. Child Care Health Dev..

[96] McCloskey M.L., Thompson D.A., Chamberlin B., Clark L., Johnson S.L., Bellows L.L. (2018). Mobile device use among rural, low-income families and the feasibility of an app to encourage preschoolers’ physical activity: Qualitative study. JMIR Pediatr. Parent..

[97] Swindle T., Poosala A.B., Zeng N., Børsheim E., Andres A., Bellows L.L. (2022). Digital intervention strategies for increasing physical activity among preschoolers: Systematic review. J. Med. Internet Res..

[98] Bai P., Thornton A., Lester L., Schipperijn J., Trapp G., Boruff B., Ng M., Wenden E., Christian H. (2020). Nature play and fundamental movement skills training programs improve childcare educator supportive physical activity behavior. Int. J. Environ. Res. Public Health.

[99] Dowdell K., Gray T., Malone K. (2011). Nature and its influence on children’s outdoor play. J. Outdoor Environ. Educ..

[100] Ayubia N., Komainib A. (2021). The Impact of the COVID-19 Pandemic on Children’s Motor Skills (Literature Review). Children.

[101] Coppens E., Rommers N., Bardid F., Deconinck F.J., De Martelaer K., D’Hondt E., Lenoir M. (2021). Long-term effectiveness of a fundamental motor skill intervention in Belgian children: A 6-year follow-up. Scand. J. Med. Sci. Sports.

[102] Making Our Move. Our Shared Vision for Uniting the Movement in Notts and Derbyshire. https://makingourmove.org.uk/about/making-our-move/.

[103] (2023). The First 1000 Days Yorkshire Giving Every Child the Best Possible Start.

[104] Lopes L., Santos R., Coelho-e-Silva M., Draper C., Mota J., Jidovt seff B., Clark C., Schmidt M., Morgan P., Duncan M. (2021). A Narrative Review of Motor Competence in Children and Adolescents: What We Know and What We Need to Find Out. Int. J. Environ. Res. Public Health.

[105] International Motor Development Research Consortium (2023). Advancing Motor Development Research in the 21st Century. https://www.i-mdrc.com/.

